# SLC14A1: a novel target for human urothelial cancer

**DOI:** 10.1007/s12094-017-1693-3

**Published:** 2017-06-06

**Authors:** R. Hou, X. Kong, B. Yang, Y. Xie, G. Chen

**Affiliations:** 1 Department of Urology, China Japan Union Hospital, Jilin University, Changchun, 130033 Jilin China; 2 Department of Pharmacology, School of Basic Medical Sciences, Peking University, Beijing, 100191 China; 3 Renal Division, Department of Medicine, Emory University School of Medicine, Atlanta, GA 30322 USA; 4 Xiangya School of Medicine, Central South University, Changsha, 410008 Hunan China; 5 Department of Physiology, Emory University School of Medicine, Whitehead Research Building Room 615, 615 Michael Street, Atlanta, GA 30322 USA

**Keywords:** Urothelium, Cancer, Urea transporter, Gene expression

## Abstract

Urinary bladder cancer is the second commonly diagnosed genitourinary malignancy. Previously, bio-molecular alterations have been observed within certain locations such as chromosome 9, retinoblastoma gene and fibroblast growth factor receptor-3. Solute carrier family 14 member 1 (SLC14A1) gene encodes the type-B urea transporter (UT-B) which facilitates the passive movement of urea across cell membrane, and has recently been related with human malignancies, especially for bladder cancer. Herein, we discussed the SLC14A1 gene and UT-B protein properties, aiming to elucidate the expression behavior of SLC14A1 in human bladder cancer. Furthermore, by reviewing some well-established theories regarding the carcinogenesis of bladder cancer, including several genome wide association researches, we have bridged the mechanisms of cancer development with the aberrant expression of SLC14A1. In conclusion, the altered expression of SLC14A1 gene in human urothelial cancer may implicate its significance as a novel target for research.

## Introduction

Urothelium is the epithelium that covers the urinary tract from renal pelvis to urethra. Human urothelial cancer may occur at any position in the urinary tract, yet with a higher frequency of existence in the urinary bladder. Urinary bladder cancer (UBC) is the second most commonly diagnosed genitourinary malignancy in the United States. It is estimated that 76,690 new cases as well as 16,390 deaths will occur in 2016 [1]. Amid various histological types, transitional cell carcinoma accounts for most of the cases [2, 3]. Approximately 70% of the non-muscle invasive transitional cell carcinoma will relapse within 5 years after the first standard transurethral resection of the bladder tumor (TURBT) [4]. Therefore, it demands intensive surveillance procedures, including long-term periodical cystoscopy screening, adjuvant intravesical chemotherapy and immunotherapy, which makes the disease one of the most expensive and suffering cancers worldwide [5]. During the past decades, significant progresses have been made in unveiling the mechanisms associated with cancer initiation, development and metastasis. Potential culprits include the deletion in chromosome 9 [6–8], point mutations of the fibroblast growth factor receptor-3 (FGFR3) [9, 10] and alterations in tumor suppressor gene TP53 and RB1 [11, 12]. However, ascribed to the repercussion of a ‘two-hit’ or even multiple hits based on the Knudson hypothesis [13], the bewildering story of cancer is far more complicated than we thought. Therefore, when the recent genome wide association studies (GWAS) revealed one of the solute carrier family gene, SLC14A1, is related to the carcinogenesis of UBC [14–17], it seems a new piece has just been added to the puzzle.

### SLC14A1 gene and UT-B protein

Human solute carrier family 14 member 1 (SLC14A1) gene contains approximately 30 kb nucleotides, and is located on chromosome 18q12.1-21.1 [18]. SLC14A1 encodes type-B urea transporter (UT-B), which resides in tandem with another urea transporter, UT-A, coded by SLC14A2 [19]. Urea transporter facilitates the rapid and passive cross-membrane movement of urea [19, 20]. Moreover, human UT-B (hUT-B) also serves as the determinant antigen of Kidd blood group on erythrocytes [19, 21]. The coding sequence of SLC14A1 consists of 11 exons [18]. So far, there have been two documented hUT-B isoforms, namely hUT-B1 and hUT-B2. hUT-B1 was first cloned from human bone marrow and shares 62.4% identity with the rabbit UT-A2 [22]. The coding sequence of hUT-B1 initiates from exon 4 and ends at exon 11, comprising 1170 nucleotides that encode a 389aa protein. It has been verified that hUT-B1 transcript exists in multiple tissues including brain, heart, lung, kidney, bladder, and prostate [23–27]. hUT-B2, however, was first identified from bovine rumen and designated as bovine UT-B2 (bUT-B2), with an additional 55-amino acid encoded by exon 3 splicing into the N-terminal of the UT-B1 sequence [28]. At first, hUT-B2 mRNA has only been identified in caudate nucleus (Genbank NM_001146037) [29]. However, recently, hUT-B2 mRNA was discovered in the human urothelium [25] (Table 1).

**Table 1:** Properties of UT-B1 and UT-B2

	Human UT-B1	Human UT-B2
Coding gene	SLC14A1 (Exon 4–11)	SLC14A1 (Exon 3–11)
Amino acids (nucleotides)	389AA (1170 bp)	445AA (1338 bp)
Glycosylation site	Asn^211^	Asn^211^
Initial isolation	Human bone marrow [22]	Bovine Rumen [28]
Tissue distribution	Brain, heart, lung, intestine, erythrocyte, kidney, bladder, prostate, testis, etc. [23–27]	Caudate nucleus (Genbank NM_001146037) [29], Bladder [25]

**Fig. 1:**
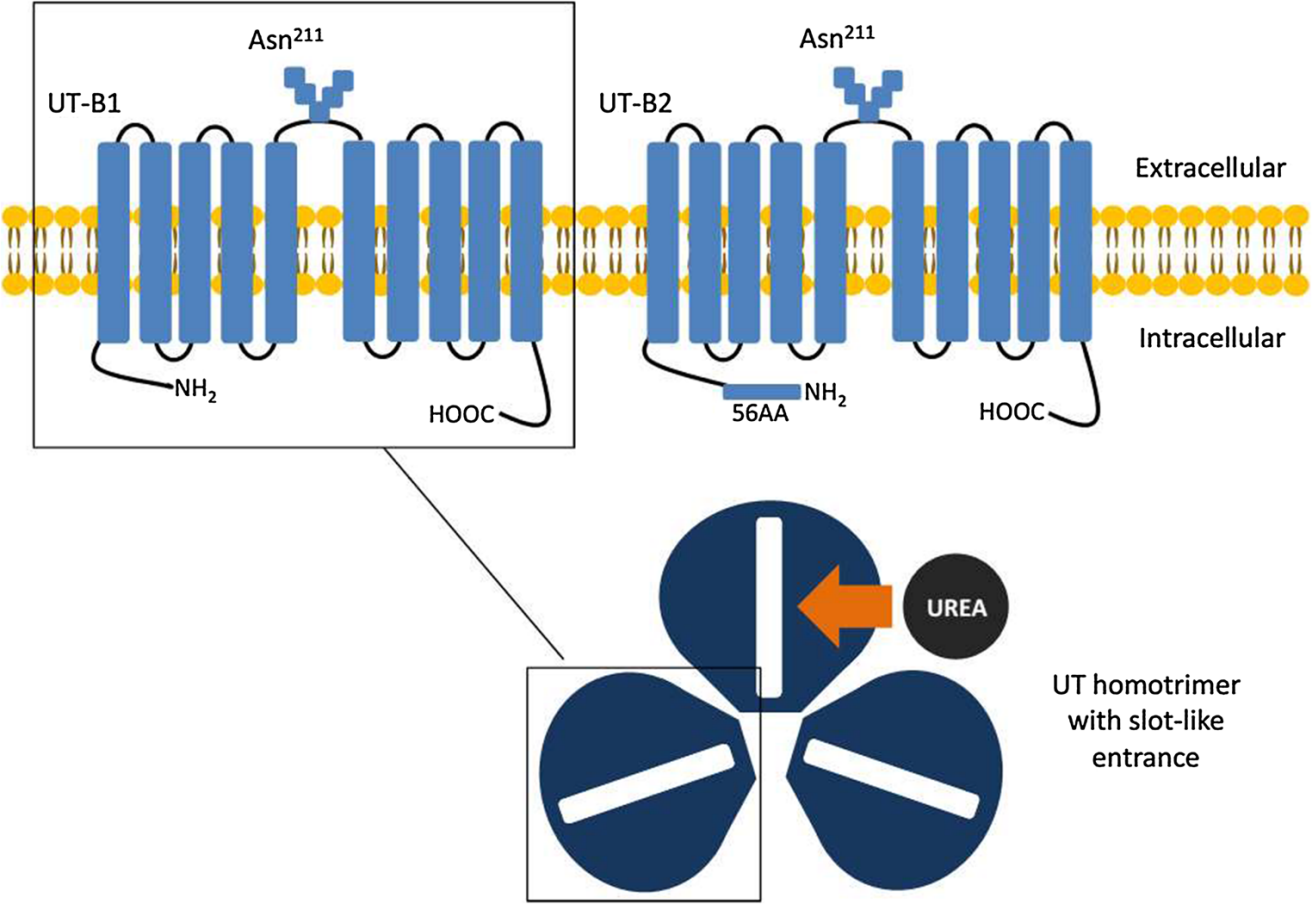
Protein structure of UT-B1 and UT-B2. Human UT-B protein contains 10 transmembrane spanning domains that are integrated into two internal hydrophobic repeats connected by a glycosylated extracellular loop, whose both amino and carboxy termini are intracellular. Asn^211^ is the only glycosylation site in human UT-B protein. The coding sequence of UT-B1 initiates from exon 4 and ends at exon 11, encoding a 389aa protein. UT-B2 has an additional 55-amino acid encoded by exon 3 splicing into the N-terminal of the UT-B1 sequence. Based on the structure theory of dvUT, urea transporter functions in the form of a homotrimer. Each promoter contains two homologous halves of the protein that has a cleft in the center. At the entrance, the parallel aromatic side chain of phenylalanine on each side forms a slot-like shape that enables only the planar urea molecule to enter

The hUT-B protein, with both intracellular amino and carboxy termini, contains ten transmembrane spanning domains that are integrated into two internal hydrophobic repeats connected by a glycosylated extracellular loop (Fig. 1) [22, 30]. Initially, there were two predicted N-glycosylation sites in the hUT-B protein when it was first cloned, Asn^211^ and Asn^291^ [22]. However, the site Asn^291^ was later proved to be unrelated with glycosylation, yet its mutation as observed in Finns did affect the transport activity and membrane expression level [31]. Therefore, Asn^211^, whose N-glycan chain also carries the ABO blood group antigens, is the only glycosylation site in hUT-B protein [31]. But the mutation of Asn^211^ neither affects the protein expression level nor its transport activity, as observed on Xenopus oocytes [32]. Another feature of hUT-B is that the protein does not conserve the potential protein kinase A (PKA) or protein kinase C (PKC) phosphorylation site as in UT-A2 [22], whereas 7 cysteine residues of UT-B are aligned at equivalent positions of UT-A2. Another 2 cysteine residues, Cys^25^ and Cys^30^, together are essential for the plasma membrane addressing, according to a mutagenesis and functional study in Xenopus oocytes [32].

When analyzed by Western blot, the glycosylated hUT-B demonstrates a 46–60 kDa smear band in erythrocytes [33] and a 41–54 kDa band in the kidney [23], both of which can be deglycosylated with peptide-*N*-glycosidase F (PNGase F) and reduced to a 32 kDa core protein [23]. Recently, a hUT-B specific signal has been detected in human bladder, which presents as a 40–45 kDa smear band that reduces to 30 kDa when deglycosylated with PNGase F [25]. Therefore, this human bladder specific UT-B is similar with that has been identified in rodents. The glycosylated urothelium UT-B is approximately 41–54 kDa in mice [34] and 35–56 kDa in rats [35], whereas the deglycosylated forms of UT-B are 29 and 32 kDa in mice and rats, respectively [34, 35]. Nevertheless, whether it is hUT-B1 or hUT-B2 that is expressed on urothelium remains a mystery, since the mRNA of both isoforms has been located in the urothelial cells. Additionally, the antibodies used in the previous researches was designed for the hUT-B C terminus, which are incapable of identifying hUT-B1 and hUT-B2 that are distinguished in the N terminus where the truncation is a part of normal physiological regulation [25, 36].

In 2009, the crystal structure of a UT homologue from the bacterium *Desulfovibrio vulgaris* (dvUT) was revealed by X-ray crystallography, which offered us a better understanding on how urea transporter works (Fig. 1) [37]. The dvUT is a homotrimer. Each promoter contains two homologous halves of the protein that has a cleft in the center. At the entrance, the parallel aromatic side chain of phenylalanine on each side forms a slot-like shape that enables only the planar urea molecule to enter [38, 39]. Inside the cleft, three linearly lined oxygen atoms constitute the bilateral oxygen ladders that continuously interact with the urea molecules via hydrogen-binding sites [37, 38]. Thus, urea molecules exhibit a stepwise movement while crossing the transporter.

Urea is a highly polarized molecule. As stated in most text books, it is freely permeable across cell membranes while the process is extremely slow [40]. Considering the transient time in which urine passes the collecting ducts of the kidney, this passive diffusion process may not be efficient enough to set up the intrarenal osmotic gradient solely and rapidly [20]. It has been observed in UT-B knock-out mice that urine urea concentration is decreased while urine output is increased [41, 42]. Actually, different types of urea transporters are expressed along the renal tubules and the vasa recta. A high urea concentration is constituted in the inner medulla collecting duct (IMCD) when urine flows through the collecting duct and water is absorbed by aquaporins. Therein, urea is reabsorbed by a vasopressin-regulated process via two types of urea transporters—UT-A1 and UT-A3 [43, 44]. The reabsorbed urea enters the ascending vasa recta (AVR) through micropores on the endothelium, and is transferred to the descending vasa recta (DVR) via UT-B subsequently [45, 46]. This process forms a countercurrent exchange and helps to preserve the urea concentration gradient in the inner medulla.

In extra-renal tissues, UT-B is believed to prevent the intracellular urea intoxication, since relevant physiology studies in UT-B null mice have observed depression-like behavior and premature of male reproductive system [24, 47]. As a urine reservoir, the bladder is constantly exposed to the high concentration of urea, which is 20–100 times higher than that of the blood [48]. Notably, UT-B exists throughout the layers of the urothelium except for the apical membrane of the umbrella cells [25]. Additionally, it has been suggested that during the process of urine replenishing and voiding, urea may enter the apical urothelial cells via the endocytic trafficking pathway [49]. Therefore, as observed in the urothelial cells of UT-B null mice, the cell cycle delay, apoptosis, and DNA damage caused by oxidative stress can be explained [50], since high urea concentration may cause the damage of DNA [51] and the disruption of the hydrophobic bonds within the protein [52]. Considering this, the abundant existence of UT-B on the bladder urothelium may imply that the potential protective role of this urea transporter.

### Molecular pathogenesis of bladder cancer

Urinary bladder cancer (UBC) is derived from the uroepithelium that covers the urinary tract from the renal pelvis to urethra. The most commonly diagnosed type of UBC is transitional cell carcinoma, which is histologically distinct from other types of UBC such as squamous cell carcinoma (related with schistosomiasis or chronic bladder irritation [53]) and adenocarcinoma (metastasized from prostate or colon [54]). Generally, UBC can be classified by their clinical behavior and the extent of cancer malignancy: non-invasive carcinoma in which cancer confines within the basement membrane (flat, papillary or inverted) and invasive UBC [55].

Papillary carcinoma (pTa UBC) arises from hyper-proliferation of the urothelium and leads to the urothelium to fold into a polyp that protrudes into the bladder. Except for papillary urothelial neoplasm of low malignant potential (PUNLMP), either low grade or high grade pTa UBC has a high tendency of recurrence [55]. The most prevalent genetic alterations reported in papillary carcinoma includes the deletions of chromosome 9, point mutations in fibroblast growth factor receptor 3 (FGFR3) and alpha catalytic subunit of phosphatidylinositol 3-kinase (PIK3CA) [7, 56] (Table 2).

**Table 2:** Well-established molecular pathways in UBC

Gene	Alterations in UBC
FGFR3 (~70%) [9]	Somatic mutation induced dimerization and auto-activation; wild-type overexpression [9, 10, 59, 60]
Chromosome 9 (~60%) [6, 8]	
CDKN2A/ARF(9p21)	Deletion and methylation [67, 68]
PTC(9q22)	Deletion [69, 70]
DBC1(9q33)	Deletion and methylation of CpG island [71–73]
TSC1(9q34)	Loss of heterozygosity [74]
PI3K (~30%, early event) [77]	PI3K/Akt pathway activation [76, 77]
P53	P53 nucleus accumulation [80]
P21^WAF^	Loss of expression [83]

FGFR3, member of the receptor tyrosine kinases family, regulates cell proliferation, differentiation and migration. The common structure of FGFR3 comprises an extracellular domain which includes three immunoglobulin (Ig) domains, a hydrophobic transmembrane region and an intracellular tyrosine kinase domain [57]. In UBC, two FGFR3-involved mechanisms may account for the tumor genesis [9, 10, 58]: first, the somatic point mutation within the FGFR3 creates a cysteine residue in the extracellular region, and gives rise to the receptor dimerization via the intermolecular disulphide bond formation followed by the ligand-independent receptor activation [59, 60]; Second, the overexpression of a wild-type receptor, which is more frequently observed in higher grade tumors. The former one is constantly associated with the low grade tumors. As observed in clinical cases, frequently mutations of FGFR3 in UBC are S249C (66.6%) and Y375C (15.1%), in exons 7 and 10, respectively [61, 62].

Deemed as one of the primary target in the carcinogenesis of UBC, chromosome 9 alterations are demonstrated in more than half of the tumors considering all grades and stages [6–8]. In previous studies, four main regions of gene deletion on chromosome 9 have been identified. On 9p21, it harbors the CDKN2A/ARF tumor suppressor gene that encodes two cell cycle regulatory proteins: cyclin-dependent kinase 2A (CDKN2A) and ARF. CDKN2A (inhibitor of CDK4) interacts with CDK4/6-cyclin D complex, maintaining the retinoblastoma (Rb) protein in its hypophosphorylated growth-suppressive form [63, 64]. The ARF, however, interacts with murine double minute 2 (MDM2), thereby inhibiting the degradation of p53 and holding the cell cycle in G1/S regulation point [65, 66]. Deletion and methylation of the CDKN2A gene inactivate both pathways, leading to an uncontrolled cell proliferation, which occurs primarily in superficial bladder tumors and is related to poor prognosis [67, 68]. On 9q22, a marker located in the first intron of the PATCHED (PTC) gene, a human ortholog of the drosophila PATCHED gene, shows the highest percentage of deletion in superficial UBC [69]. In an animal research, BBN (*N*-butyl-*N*-(4-hydroxybutyl) nitrosamine) induced bladder preneoplastic and neoplastic changes were observed significantly earlier in the PTC^+/−^ mice comparing to wild-type, suggesting that the PTC might act as a tumor suppressor in UBC [70]. In addition, within the DBC1 (deleted in bladder cancer 1) gene on 9q33, occasional homozygous deletion and methylation in CpG island have been reported in several studies [71–73]. Another gene that shows loss of heterozygosity in more than 50% of the transitional cell carcinomas is the tuberous sclerosis complex 1 (TSC1) on 9q34. The missense mutations of TSC1 were identified in 14.5% of the tumors, which causes the disfunction of TSC1 by aberrant splicing or reduced protein stability [74].

Phosphoinositide-3 kinase (PI3K) catalytic unit p110 alpha (PI3KCA) interacts with the Ras protein in a GTP-dependent manner, leading to the activation of PI3K/protein kinase B (Akt) pathway [75]. The PI3K/Akt signaling pathway, which demonstrates a prevalent activation in the entire spectrum of UBC, is considered to play a major role in carcinogenesis. In T1 and T2 UBC, high frequency of PI3KCA gene alteration has been observed. However, the presence of PI3KCA gene alteration is significantly associated with reduced recurrence of non-muscle invasive UBC [76, 77].

Invasive UBC, which generates from the flat dysplasia that leads to the CIS, comprises the tumors invading through the lamina propria into the muscularis of, or beyond, the bladder wall. Generally speaking, invasive bladder tumors frequently show alterations in p53 and Rb pathways [12, 78]. p53 is the most commonly mutated genes in human cancer, including UBC. Missense point mutations as well as the loss of a single TP53 gene allele lead to the p53 protein resistance to normal regulatory degradation by ubiquitin pathway and accumulation in the nucleus [79]. It has been observed that the accumulation of inefficient p53 in the nucleus is correlated with a worse pathological outcome, increased risk of recurrence and decreased overall survival rate [80]. p21^WAF1^ is an important downstream target of the p53 pathway. p21^WAF1^ acts as a cyclin-dependent kinase and regulates the G1-S-phase transition in the cell cycle [81, 82]. Loss of p21^WAF1^ expression is an independent predictor of UBC progression. Maintenance of its expression tends to counteract the deleterious effects of p53 alterations on UBC progression [83]. Meanwhile, alterations in both Rb and p53 pathway have been observed to act in a cooperative manner to promote cancer progression [11].

### SLC14A1 and UBC, a complicated story

In 2011, Frullanti identified the down-regulated expression of SLC14A1 in lung adenocarcinoma (ADCA) specimens and A549 (ADCA) cell lines. Meanwhile, they also discovered that transfecting the NCI-H520 (lung squamous cell carcinoma) cell line with the SLC14A1 gene significantly inhibited the colony formation [27]. In Markku’s research, SLC14A1 gene was found down-regulated by 2.88-fold in the malignant prostate cancer tissues compared with the benign ones using a genechip assay. Castration, however, elevated SLC14A1 expression by 3.05-fold, indicating that the expression of SLC14A1 gene in prostate could be regulated by androgen [26]. Meanwhile coincidentally, the expression level of SLC14A1 is also indentified to be suppressed in UBC, which, more importantly, is inversely proportional with the clinical grade and stage [84].

So far, GWAS researches have revealed several suspicious SNPs (single nucleotide polymorphism) within the SLC14A1 gene that are strongly associated with bladder cancer, such as rs1058396, rs2298720, rs11877062 and rs17674580 [14–17]. Accordingly, the G allele at nucleotide 838 of rs1058396 which encodes Asp280 defining Jk(A) in the Kidd blood system turns out to be a risk allele, and the transition from G to A (Asn280), which encodes Jk(B), tends to be a protective allele [14]. Another protective allele indicated by GWAS is rs2298720 [14]. The non-synonymous variant rs2298720 (Glu44Lys) defines a weaker Jk(A) antigen-Jk(A)W. Compared to its wild type, the Jk(A)W UT-B has a Val-Gly triplicate after Pro227 and demonstrates a weaker signal when transfected and expressed on the membrane of the Xenopus oocyte [85]. However, even though the in vitro experiment has demonstrated that the urea transport facilitated by JK(A)W is less effective than JK(A) [85], there is no direct evidence currently indicating the differences in renal function among the people bearing JK(A), JK(B) and JK(A)W [14]. In 2013, Koutros et al. discovered that people bearing another risk SLC14A1 allele, rs10775480, manifest a decreased urine specific gravity, which was independent of urination frequency and urine output [86]. Therefore, the urinary bladder, or human urothelium to be specific, could play an important role in the urinary solute regulation, just as previously described by Dr. Apodaca [49], and such regulatory malfunction could be the culprit for the development of UBC.

In the UT-B knock-out mice, the ‘urea scavenger’ deficiency has caused severe apoptosis and DNA damage in the urothelial cells where the urea concentration is nine times higher than that of the wild type [50]. This devastating phenomenon possibly caused by urea accumulation coincides with a previous study, in which high urea concentration had caused cell cycle delay in G2/M and G0/G1 phase as well as the apoptotic cell death [51]. Based on the chemical reaction of Wöhler synthesis discovered in 1828, urea can spontaneously transform into cyanate and ammonia at body temperature and pH [87]. Cyanate then converts free amino acids into carbamoyl amino acids, which can in turn interfere with protein synthesis [88]. Additionally, urea can also destabilize protein by decreasing the hydrophobic effect and directly binding with the amide groups through hydrogen bond [52]. In addition, plasma urea concentration of UBC patients was observed to be significantly elevated [89]. Therefore, the suppressed expression of hUT-B in the background of UBC could possibly lead to the urea accumulation within the urothelial cells, which subsequently induces the generation of cytotoxic reagents, severe protein and DNA damage, and the eventual apoptosis. Intriguingly, cancer development in which proliferation is frequently involved is divergent from programmed cell death in many ways. However, it has been reported that during the process of apoptosis, receptors such as FAS and TNF-related apoptosis-inducing ligand receptor (TRAILR) may present non-apoptotic functions, including proliferation and invasion, which can possibly induce the development of cancer [90, 91]. Thus, in the future researches, the function of TRAILR in UT-B knock-out models should be investigated.

**Fig. 2:**
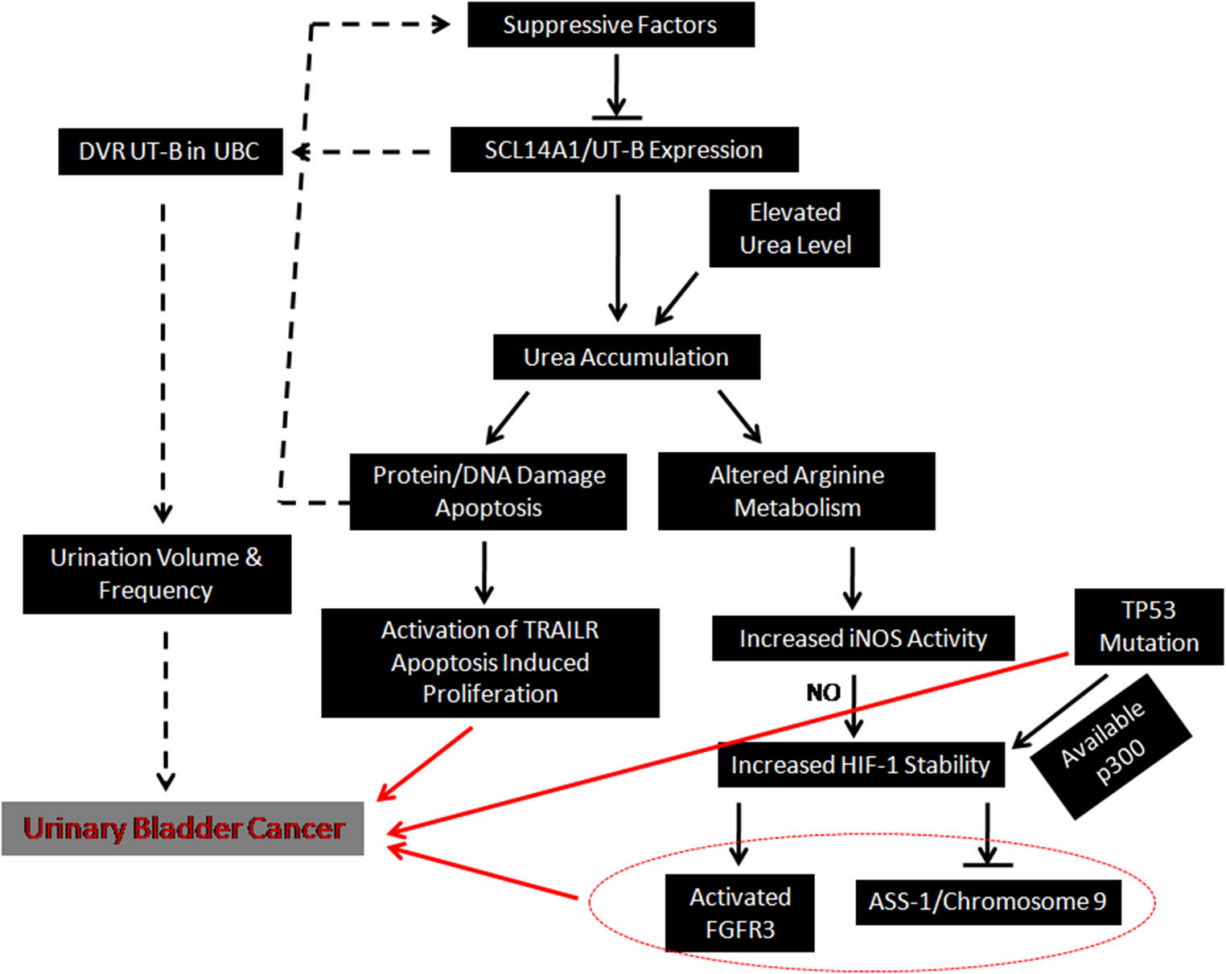
Illustration of the relationship between SLC14A1 and UBC. UT-B may act as a tumor suppressor that is somehow down-regulated by suppressive factors, inducing intracellular urea accumulation. In addition to the elevated plasma urea level, intracellular urea overload can cause protein/DNA damage and trigger and apoptosis. Subsequently, the apoptosis may induce the activation of TRAILR and the initiation of cancer. On the other hand, urea accumulation could alter the intracellular arginine metabolism, which activates HIF-1 via NO. HIF-1 may interact with canonical UBC pathways including FGFR3, chromosome 9 and p53. Notably, the DVR UT-B expression in the background of UBC probably needs more attention in the future research, since it serves to regulate urine volume and frequency, which can be the causes for urothelium neoplasms

Another effect of urea accumulation inside the urothelium is the alteration of l-arginine metabolism, which increases the intracellular level of inducible NO synthase (iNOS) [50]. As a downstream target, hypoxia-inducible factor-1 (HIF-1) is stabilized by the high concentration of nitric oxide (NO) synthesized via iNOS catalyzing [92, 93]. Consequently, the sequestration of urea within the urothelial cells stabilizes HIF-1, a negative regulator of argininosuccinate synthetase 1 (ASS-1) [94]. The gene encodes ASS-1 is localized on chromosome 9q34. It has been reported that the ASS-1 may act as a cancer suppressor, and was lost in approximately 40% of the UBC, primarily caused by the transcriptional suppression induced by HIF-1 or the well-known chromosome 9 deletion [8, 95]. Meanwhile, HIF-1 also competes with p53 for p300, a transcriptional activator [96]. In concomitance with the inability of p53 caused by genetic mutation, the HIF-1 becomes the dominantly activated by p300 [97], and is also identified to enhance the expression level of FGFR3 in non-muscle invasive UBC [98]. Therefore, it seems that the urea accumulation induced by hUT-B dysfunction in the urothelium may trigger intracellular metabolic disorders that could interact with canonical UBC pathways (Fig. 2).

Hence, it could be postulated that UT-B might act as a tumor suppressor that is somehow down-regulated, inducing intracellular urea accumulation which in turn causes DNA damage and triggering the initiation of cancer via multiple pathways. However, whether such down-regulation of UT-B is an universal event that could impair its capacity in orchestrating urine volume and frequency remains unclear. It has been reported that urination frequency and volume could be the potential causes for urothelium neoplasms [99, 100]. Therefore, further research should consider the urothelial UT-B and the DVR UT-B wholly when evaluating the association between SLC14A1 gene and UBC (Fig. 2).

## Conclusion

The alternative expression of SLC14A1 in human UBC has been observed in several studies, including large population-based GWAS researches. Based on the literatures in this review, we conclude that the impaired expression of UT-B in human urothelial cells could lead to urea intracellular accumulation and subsequent metabolic disorders. Accordingly, we postulated that two potential downstream pathways could be involved in the carcinogenesis under such circumstances, including apoptosis-induced proliferation that activated by TRAILR, and NO triggered HIF-1 promoted oncogene expression and tumor suppressor gene down-regulation. However, more comprehensive investigations are needed to elucidate the underlying mechanisms that initially caused the repression of SLC14A1 in UBC, and to unveil the relationship between aberrant SLC14A1 expression and the carcinogenesis of UBC. Additionally, the cooperation of renal and extra-renal UT-B in the development of UBC should be considered as well, since urination frequency and volume may also play an important role. In sum, SLC14A1 and UT-B should be a novel and promising research target in the field of urothelial cancer.

## References

[1] American Cancer Society (2016). Cancer facts and figures 2016.

[2] Karaoglu I, van der Heijden AG, Witjes JA (2014). The role of urine markers, white light cystoscopy and fluorescence cystoscopy in recurrence, progression and follow-up of non-muscle invasive bladder cancer. World J Urol.

[3] Fadl-Elmula I, Gorunova L, Mandahl N, Elfving P, Lundgren R, Mitelman F, Heim S (2000). Karyotypic characterization of urinary bladder transitional cell carcinomas. Genes Chromosomes Cancer.

[4] Chamie K, Litwin MS, Bassett JC, Daskivich TJ, Lai J, Hanley JM, Konety BR, Saigal CS (2013). Urologic Diseases in America Project. Recurrence of high-risk bladder cancer: a population-based analysis. Cancer.

[5] Svatek RS, Hollenbeck BK, Holmäng S, Lee R, Kim SP, Stenzl A, Lotan Y (2014). The economics of bladder cancer: costs and considerations of caring for this disease. Eur Urol.

[6] Knowles MA (2008). Molecular pathogenesis of bladder cancer. Int J Clin Oncol..

[7] Schulz WA (2006). Understanding urothelial carcinoma through cancer pathways. Int J Cancer.

[8] Miyao N, Tsai YC, Lerner SP, Olumi AF, Spruck CH, Gonzalez-Zulueta M, Nichols PW, Skinner DG, Jones PA (1993). Role of chromosome 9 in human bladder cancer. Cancer Res.

[9] Iyer G, Milowsky MI (2013). Fibroblast growth factor receptor-3 in urothelial tumorigenesis. Urol Oncol..

[10] Pandith AA, Shah ZA, Siddiqi MA (2013). Oncogenic role of fibroblast growth factor receptor 3 in tumorigenesis of urinary bladder cancer. Urol Oncol..

[11] Cote RJ, Dunn MD, Chatterjee SJ, Stein JP, Shi SR, Tran QC, Hu SX, Xu HJ, Groshen S, Taylor CR, Skinner DG, Benedict WF (1998). Elevated and absent pRb expression is associated with bladder cancer progression and has cooperative effects with p53. Cancer Res.

[12] Mitra AP, Datar RH, Cote RJ (2006). Molecular pathways in invasive bladder cancer: new insights into mechanisms, progression, and target identification. J Clin Oncol.

[13] Knudson AG (1971). Mutation and cancer: statistical study of retinoblastoma. Proc Natl Acad Sci USA..

[14] Garcia-Closas M, Ye Y, Rothman N, Figueroa JD, Malats N, Dinney CP, Chatterjee N, Prokunina-Olsson L, Wang Z, Lin J, Real FX, Jacobs KB, Baris D, Thun M, De Vivo I, Albanes D, Purdue MP, Kogevinas M, Kamat AM, Lerner SP, Grossman HB, Gu J, Pu X, Hutchinson A, Fu YP, Burdett L, Yeager M, Tang W, Tardón A, Serra C, Carrato A, García-Closas R, Lloreta J, Johnson A, Schwenn M, Karagas MR, Schned A, Andriole G, Grubb R, Black A, Jacobs EJ, Diver WR, Gapstur SM, Weinstein SJ, Virtamo J, Hunter DJ, Caporaso N, Landi MT, Fraumeni JF, Silverman DT, Chanock SJ, Wu X (2011). A genome-wide association study of bladder cancer identifies a new susceptibility locus within SLC14A1, a urea transporter gene on chromosome 18q12.3. Hum Mol Genet.

[15] Rafnar T, Vermeulen SH, Sulem P, Thorleifsson G, Aben KK, Witjes JA, Grotenhuis AJ, Verhaegh GW, Hulsbergen-van de Kaa CA, Besenbacher S, Gudbjartsson D, Stacey SN, Gudmundsson J, Johannsdottir H, Bjarnason H, Zanon C, Helgadottir H, Jonasson JG, Tryggvadottir L, Jonsson E, Geirsson G, Nikulasson S, Petursdottir V, Bishop DT, Chung-Sak S, Choudhury A, Elliott F, Barrett JH, Knowles MA, de Verdier PJ, Ryk C, Lindblom A, Rudnai P, Gurzau E, Koppova K, Vineis P, Polidoro S, Guarrera S, Sacerdote C, Panadero A, Sanz-Velez JI, Sanchez M, Valdivia G, Garcia-Prats MD, Hengstler JG, Selinski S, Gerullis H, Ovsiannikov D, Khezri A, Aminsharifi A, Malekzadeh M, van den Berg LH, Ophoff RA, Veldink JH, Zeegers MP, Kellen E, Fostinelli J, Andreoli D, Arici C, Porru S, Buntinx F, Ghaderi A, Golka K, Mayordomo JI, Matullo G, Kumar R, Steineck G, Kiltie AE, Kong A, Thorsteinsdottir U, Stefansson K, Kiemeney LA (2011). European genome-wide association study identifies SLC14A1 as a new urinary bladder cancer susceptibility gene. Hum Mol Genet.

[16] Singh V, Jaiswal PK, Mittal RD (2014). Replicative study of GWAS TP63C/T, TERTC/T, and SLC14A1C/T with susceptibility to bladder cancer in North Indians. Urol Oncol..

[17] Matsuda K, Takahashi A, Middlebrooks CD, Obara W, Nasu Y, Inoue K, Tamura K, Yamasaki I, Naya Y, Tanikawa C, Cui R, Figueroa JD, Silverman DT, Rothman N, Namiki M, Tomita Y, Nishiyama H, Kohri K, Deguchi T, Nakagawa M, Yokoyama M, Miki T, Kumon H, Fujioka T, Prokunina-Olsson L, Kubo M, Nakamura Y, Shuin T (2015). Genome-wide association study identified SNP on 15q24 associated with bladder cancer risk in Japanese population. Hum Mol Genet.

[18] Lucien N, Sidoux-Walter F, Olivès B, Moulds J, Le Pennec PY, Cartron JP, Bailly P (1998). Characterization of the gene encoding the human Kidd blood group/urea transporter protein. Evidence for splice site mutations in Jknull individuals. J Biol Chem.

[19] Shayakul C, Hediger MA (2004). The SLC14 gene family of urea transporters. Pflugers Arch.

[20] Sands JM (2003). Molecular mechanisms of urea transport. J Membr Biol.

[21] Yang B (2014). Transport characteristics of urea transporter-B. Subcell Biochem.

[22] Olives B, Neau P, Bailly P, Hediger MA, Rousselet G, Cartron JP, Ripoche P (1994). Cloning and functional expression of a urea transporter from human bone marrow cells. J Biol Chem.

[23] Timmer RT, Klein JD, Bagnasco SM, Doran JJ, Verlander JW, Gunn RB, Sands JM (2001). Localization of the urea transporter UT-B protein in human and rat erythrocytes and tissues. Am J Physiol Cell Physiol.

[24] Li X, Ran J, Zhou H, Lei T, Zhou L, Han J, Yang B (2012). Mice lacking urea transporter UT-B display depression-like behavior. J Mol Neurosci.

[25] Walpole C, Farrell A, McGrane A, Stewart GS (2014). Expression and localization of a UT-B urea transporter in the human bladder. Am J Physiol Renal Physiol.

[26] Vaarala MH, Hirvikoski P, Kauppila S, Paavonen TK (2012). Identification of androgen-regulated genes in human prostate. Mol Med Rep..

[27] Frullanti E, Colombo F, Falvella FS, Galvan A, Noci S, De Cecco L, Incarbone M, Alloisio M, Santambrogio L, Nosotti M, Tosi D, Pastorino U, Dragani TA (2012). Association of lung adenocarcinoma clinical stage with gene expression pattern in noninvolved lung tissue. Int J Cancer.

[28] Stewart GS, Graham C, Cattell S, Smith TP, Simmons NL, Smith CP (2005). UT-B is expressed in bovine rumen: potential role in ruminal urea transport. Am J Physiol Regul Integr Comp Physiol.

[29] Stewart G (2011). The emerging physiological roles of the SLC14A family of urea transporters. Br J Pharmacol.

[30] Olivès B, Martial S, Mattei MG, Matassi G, Rousselet G, Ripoche P, Cartron JP, Bailly P (1996). Molecular characterization of a new urea transporter in the human kidney. FEBS Lett.

[31] Sidoux-Walter F, Lucien N, Nissinen R, Sistonen P, Henry S, Moulds J, Cartron JP, Bailly P (2000). Molecular heterogeneity of the Jk(null) phenotype: expression analysis of the Jk(S291P) mutation found in Finns. Blood.

[32] Lucien N, Sidoux-Walter F, Roudier N, Ripoche P, Huet M, Trinh-Trang-Tan MM, Cartron JP, Bailly P (2002). Antigenic and functional properties of the human red blood cell urea transporter hUT-B1. J Biol Chem.

[33] Olivès B, Mattei MG, Huet M, Neau P, Martial S, Cartron JP, Bailly P (1995). Kidd blood group and urea transport function of human erythrocytes are carried by the same protein. J Biol Chem.

[34] Lucien N, Bruneval P, Lasbennes F, Belair MF, Mandet C, Cartron J, Bailly P, Trinh-Trang-Tan MM (2005). UT-B1 urea transporter is expressed along the urinary and gastrointestinal tracts of the mouse. Am J Physiol Regul Integr Comp Physiol.

[35] Spector DA, Yang Q, Liu J, Wade JB (2004). Expression, localization, and regulation of urea transporter B in rat urothelia. Am J Physiol Renal Physiol.

[36] Sands JM, Blount MA (2014). Genes and proteins of urea transporters. Subcell Biochem.

[37] Levin EJ, Quick M, Zhou M (2009). Crystal structure of a bacterial homologue of the kidney urea transporter. Nature.

[38] Levin EJ, Cao Y, Enkavi G, Quick M, Pan Y, Tajkhorshid E, Zhou M (2012). Structure and permeation mechanism of a mammalian urea transporter. Proc Natl Acad Sci USA..

[39] Knepper MA, Mindell JA (2009). Structural biology: molecular coin slots for urea. Nature.

[40] Gallucci E, Micelli S, Lippe C (1971). Non-electrolyte permeability across thin lipid membranes. Arch Int Physiol Biochim..

[41] Yang B, Bankir L, Gillespie A, Epstein CJ, Verkman AS (2002). Urea-selective concentrating defect in transgenic mice lacking urea transporter UT-B. J Biol Chem.

[42] Li X, Chen G, Yang B (2012). Urea transporter physiology studied in knockout mice. Front Physiol..

[43] Klein JD, Fröhlich O, Blount MA, Martin CF, Smith TD, Sands JM (2006). Vasopressin increases plasma membrane accumulation of urea transporter UT-A1 in rat inner medullary collecting ducts. J Am Soc Nephrol.

[44] Cai Q, Nelson SK, McReynolds MR, Diamond-Stanic MK, Elliott D, Brooks HL (2010). Vasopressin increases expression of UT-A1, UT-A3, and ER chaperone GRP78 in the renal medulla of mice with a urinary concentrating defect. Am J Physiol Renal Physiol.

[45] Klein JD, Blount MA, Sands JM (2012). Molecular mechanisms of urea transport in health and disease. Pflugers Arch.

[46] Klein JD, Blount MA, Sands JM (2011). Urea transport in the kidney. Compr Physiol..

[47] Guo L, Zhao D, Song Y, Meng Y, Zhao H, Zhao X, Yang B (2007). Reduced urea flux across the blood-testis barrier and early maturation in the male reproductive system in UT-B-null mice. Am J Physiol Cell Physiol.

[48] Yang B, Bankir L (2005). Urea and urine concentrating ability: new insights from studies in mice. Am J Physiol Renal Physiol.

[49] Apodaca G (2004). The uroepithelium: not just a passive barrier. Traffic..

[50] Dong Z, Ran J, Zhou H, Chen J, Lei T, Wang W, Sun Y, Lin G, Bankir L, Yang B (2013). Urea transporter UT-B deletion induces DNA damage and apoptosis in mouse bladder urothelium. PLoS One.

[51] Michea L, Ferguson DR, Peters EM, Andrews PM, Kirby MR, Burg MB (2000). Cell cycle delay and apoptosis are induced by high salt and urea in renal medullary cells. Am J Physiol Renal Physiol.

[52] Zou Q, Habermann-Rottinghaus SM, Murphy KP (1998). Urea effects on protein stability: hydrogen bonding and the hydrophobic effect. Proteins..

[53] Martin JW, Carballido EM, Ahmed A, Farhan B, Dutta R, Smith C, Youssef RF (2016). Squamous cell carcinoma of the urinary bladder: systematic review of clinical characteristics and therapeutic approaches. Arab J Urol..

[54] Dadhania V, Czerniak B, Guo CC (2015). Adenocarcinoma of the urinary bladder. Am J Clin Exp Urol..

[55] Amin MB, McKenney JK, Paner GP, Hansel DE, Grignon DJ, Montironi R, Lin O, Jorda M, Jenkins LC, Soloway M, Epstein JI, Reuter VE (2013). International Consultation on Urologic Disease-European Association of Urology Consultation on Bladder Cancer 2012. ICUD-EAU International Consultation on Bladder Cancer 2012: pathology. Eur Urol.

[56] Mitra AP, Cote RJ (2009). Molecular pathogenesis and diagnostics of bladder cancer. Annu Rev Pathol.

[57] Böttcher RT, Niehrs C (2005). Fibroblast growth factor signaling during early vertebrate development. Endocr Rev.

[58] Knowles MA (2007). Role of FGFR3 in urothelial cell carcinoma: biomarker and potential therapeutic target. World J Urol.

[59] Webster MK, Donoghue DJ (1997). Enhanced signaling and morphological transformation by a membrane-localized derivative of the fibroblast growth factor receptor 3 kinase domain. Mol Cell Biol.

[60] Tomlinson DC, Hurst CD, Knowles MA (2007). Knockdown by shRNA identifies S249C mutant FGFR3 as a potential therapeutic target in bladder cancer. Oncogene.

[61] Bernard-Pierrot I, Brams A, Dunois-Lardé C, Caillault A, Diez de Medina SG, Cappellen D, Graff G, Thiery JP, Chopin D, Ricol D, Radvanyi F (2006). Oncogenic properties of the mutated forms of fibroblast growth factor receptor 3b. Carcinogenesis.

[62] Rieger-Christ KM, Mourtzinos A, Lee PJ, Zagha RM, Cain J, Silverman M, Libertino JA, Summerhayes IC (2003). Identification of fibroblast growth factor receptor 3 mutations in urine sediment DNA samples complements cytology in bladder tumor detection. Cancer.

[63] Weinberg RA (1995). The retinoblastoma protein and cell cycle control. Cell.

[64] Serrano M, Hannon GJ, Beach D (1993). A new regulatory motif in cell-cycle control causing specific inhibition of cyclin D/CDK4. Nature.

[65] Dominguez-Brauer C, Brauer PM, Chen YJ, Pimkina J, Raychaudhuri P (2010). Tumor suppression by ARF: gatekeeper and caretaker. Cell Cycle.

[66] Inoue K, Fry EA, Frazier DP (2016). Transcription factors that interact with p53 and Mdm2. Int J Cancer.

[67] Orlow I, LaRue H, Osman I, Lacombe L, Moore L, Rabbani F, Meyer F, Fradet Y, Cordon-Cardo C (1999). Deletions of the INK4A gene in superficial bladder tumors. Association with recurrence. Am J Pathol..

[68] Berggren P, Kumar R, Sakano S, Hemminki L, Wada T, Steineck G, Adolfsson J, Larsson P, Norming U, Wijkström H, Hemminki K (2003). Detecting homozygous deletions in the CDKN2A(p16(INK4a))/ARF(p14(ARF)) gene in urinary bladder cancer using real-time quantitative PCR. Clin Cancer Res.

[69] Aboulkassim TO, LaRue H, Lemieux P, Rousseau F, Fradet Y (2003). Alteration of the PATCHED locus in superficial bladder cancer. Oncogene.

[70] Hamed S, LaRue H, Hovington H, Girard J, Jeannotte L, Latulippe E, Fradet Y (2004). Accelerated induction of bladder cancer in patched heterozygous mutant mice. Cancer Res.

[71] Nishiyama H, Takahashi T, Kakehi Y, Habuchi T, Knowles MA (1999). Homozygous deletion at the 9q32-33 candidate tumor suppressor locus in primary human bladder cancer. Genes Chromosomes Cancer.

[72] Habuchi T, Luscombe M, Elder PA, Knowles MA (1998). Structure and methylation-based silencing of a gene (DBCCR1) within a candidate bladder cancer tumor suppressor region at 9q32-q33. Genomics.

[73] Salem C, Liang G, Tsai YC, Coulter J, Knowles MA, Feng AC, Groshen S, Nichols PW, Jones PA (2000). Progressive increases in de novo methylation of CpG islands in bladder cancer. Cancer Res.

[74] Pymar LS, Platt FM, Askham JM, Morrison EE, Knowles MA (2008). Bladder tumour-derived somatic TSC1 missense mutations cause loss of function via distinct mechanisms. Hum Mol Genet.

[75] Ching CB, Hansel DE (2010). Expanding therapeutic targets in bladder cancer: the PI3K/Akt/mTOR pathway. Lab Invest.

[76] Calderaro J, Rebouissou S, de Koning L, Masmoudi A, Hérault A, Dubois T, Maille P, Soyeux P, Sibony M, de la Taille A, Vordos D, Lebret T, Radvanyi F, Allory Y (2014). PI3K/AKT pathway activation in bladder carcinogenesis. Int J Cancer.

[77] Dueñas M, Martínez-Fernández M, García-Escudero R, Villacampa F, Marqués M, Saiz-Ladera C, Duarte J, Martínez V, Gómez MJ, Martín ML, Fernández M, Castellano D, Real FX, Rodriguez-Peralto JL, De La Rosa F, Paramio JM (2015). PIK3CA gene alterations in bladder cancer are frequent and associate with reduced recurrence in non-muscle invasive tumors. Mol Carcinog.

[78] Raghavan D (2003). Molecular targeting and pharmacogenomics in the management of advanced bladder cancer. Cancer.

[79] Dalbagni G, Presti JC, Reuter VE, Zhang ZF, Sarkis AS, Fair WR, Cordon-Cardo C (1993). Molecular genetic alterations of chromosome 17 and p53 nuclear overexpression in human bladder cancer. Diagn Mol Pathol.

[80] Esrig D, Elmajian D, Groshen S, Freeman JA, Stein JP, Chen SC, Nichols PW, Skinner DG, Jones PA, Cote RJ (1994). Accumulation of nuclear p53 and tumor progression in bladder cancer. N Engl J Med.

[81] Livingstone LR, White A, Sprouse J, Livanos E, Jacks T, Tlsty TD (1992). Altered cell cycle arrest and gene amplification potential accompany loss of wild-type p53. Cell.

[82] El-Deiry WS, Tokino T, Velculescu VE, Levy DB, Parsons R, Trent JM, Lin D, Mercer WE, Kinzler KW, Vogelstein B (1993). WAF1, a potential mediator of p53 tumor suppression. Cell..

[83] Stein JP, Ginsberg DA, Grossfeld GD, Chatterjee SJ, Esrig D, Dickinson MG, Groshen S, Taylor CR, Jones PA, Skinner DG, Cote RJ (1998). Effect of p21WAF1/CIP1 expression on tumor progression in bladder cancer. J Natl Cancer Inst.

[84] Li C, Xue H, Lei Y, Zhu J, Yang B, Gai X (2014). Clinical significance of the reduction of UT-B expression in urothelial carcinoma of the bladder. Pathol Res Pract.

[85] Sidoux-Walter F, Lucien N, Olivès B, Gobin R, Rousselet G, Kamsteeg EJ, Ripoche P, Deen PM, Cartron JP, Bailly P (1999). At physiological expression levels the Kidd blood group/urea transporter protein is not a water channel. J Biol Chem.

[86] Koutros S, Baris D, Fischer A, Tang W, Garcia-Closas M, Karagas MR, Schwenn M, Johnson A, Figueroa J, Waddell R, Prokunina-Olsson L, Rothman N, Silverman DT (2013). Differential urinary specific gravity as a molecular phenotype of the bladder cancer genetic association in the urea transporter gene, SLC14A1. Int J Cancer.

[87] Dirnhuber P, Schütz F (1948). The isomeric transformation of urea into ammonium cyanate in aqueous solutions. Biochem J..

[88] Kraus LM, Kraus AP (2001). Carbamoylation of amino acids and proteins in uremia. Kidney Int Suppl.

[89] Lattermann R, Geisser W, Georgieff M, Wachter U, Goertz A, Gnann R, Schricker T (2003). Integrated analysis of glucose, lipid, and urea metabolism in patients with bladder cancer. Impact of tumor stage. Nutrition..

[90] Ichim G, Tait SW (2016). A fate worse than death: apoptosis as an oncogenic process. Nat Rev Cancer.

[91] Ryoo HD, Bergmann A (2012). The role of apoptosis-induced proliferation for regeneration and cancer. Cold Spring Harb Perspect Biol..

[92] Mateo J, García-Lecea M, Cadenas S, Hernández C, Moncada S (2003). Regulation of hypoxia-inducible factor-1 alpha by nitric oxide through mitochondria-dependent and -independent pathways. Biochem J..

[93] Ortiz-Masiá D, Hernández C, Quintana E, Velázquez M, Cebrián S, Riaño A, Calatayud S, Esplugues JV, Barrachina MD (2010). iNOS-derived nitric oxide mediates the increase in TFF2 expression associated with gastric damage: role of HIF-1. FASEB J..

[94] Haines RJ, Pendleton LC, Eichler DC (2011). Argininosuccinate synthase: at the center of arginine metabolism. Int J Biochem Mol Biol..

[95] Allen MD, Luong P, Hudson C, Leyton J, Delage B, Ghazaly E, Cutts R, Yuan M, Syed N, Lo Nigro C, Lattanzio L, Chmielewska-Kassassir M, Tomlinson I, Roylance R, Whitaker HC, Warren AY, Neal D, Frezza C, Beltran L, Jones LJ, Chelala C, Wu BW, Bomalaski JS, Jackson RC, Lu YJ, Crook T, Lemoine NR, Mather S, Foster J, Sosabowski J, Avril N, Li CF, Szlosarek PW (2014). Prognostic and therapeutic impact of argininosuccinate synthetase 1 control in bladder cancer as monitored longitudinally by PET imaging. Cancer Res.

[96] Schmid T, Zhou J, Köhl R, Brüne B (2004). p300 relieves p53-evoked transcriptional repression of hypoxia-inducible factor-1 (HIF-1). Biochem J..

[97] Schmid T, Zhou J, Brüne B (2004). HIF-1 and p53: communication of transcription factors under hypoxia. J Cell Mol Med.

[98] Blick C, Ramachandran A, Wigfield S, McCormick R, Jubb A, Buffa FM, Turley H, Knowles MA, Cranston D, Catto J, Harris AL (2013). Hypoxia regulates FGFR3 expression via HIF-1α and miR-100 and contributes to cell survival in non-muscle invasive bladder cancer. Br J Cancer.

[99] Jiang X, Castelao JE, Groshen S, Cortessis VK, Shibata DK, Conti DV, Gago-Dominguez M (2008). Water intake and bladder cancer risk in Los Angeles County. Int J Cancer.

[100] Villanueva CM, Cantor KP, King WD, Jaakkola JJ, Cordier S, Lynch CF, Porru S, Kogevinas M (2006). Total and specific fluid consumption as determinants of bladder cancer risk. Int J Cancer.

